# Ripped, Shucked, and Scattered

**DOI:** 10.3201/eid1908.AC1908

**Published:** 2013-08

**Authors:** Polyxeni Potter

**Affiliations:** Centers for Disease Control and Prevention, Atlanta, Georgia, USA

**Keywords:** art science connection, emerging infectious diseases, art and medicine, Catherine M. Howell, Ripped, Shucked, and Scattered, Oyster Shuckers, raw seafood consumption, norovirus infections, enteric diseases, about the cover

**Figure:**
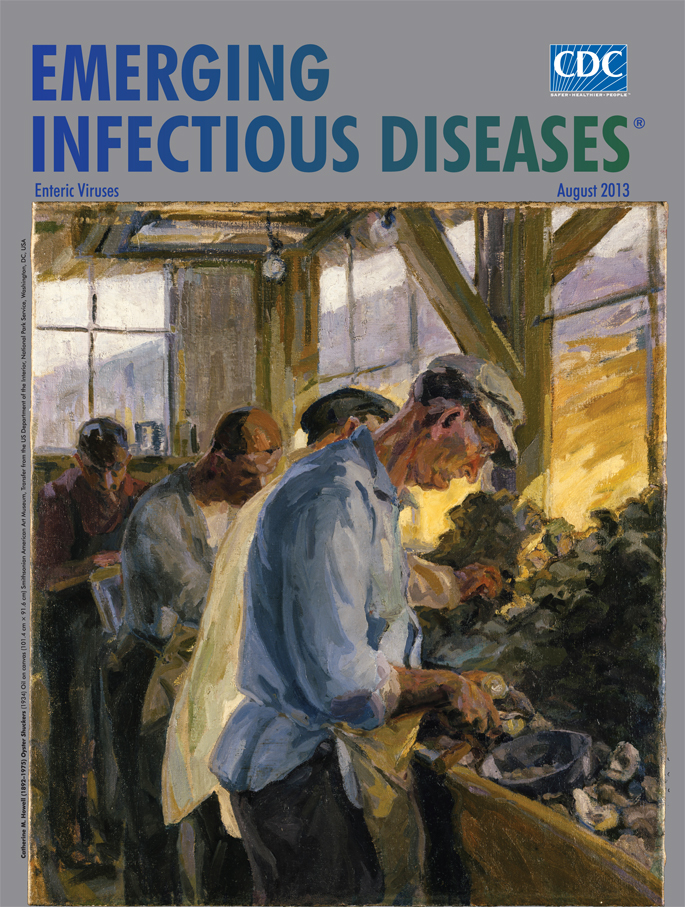
**Catherine M. Howell (1892–1975) *Oyster Shuckers* (1934) OiI on canvas (101.4 cm × 91.6 cm)** Smithsonian American Art Museum, Transfer from the US Department of the Interior, National Park Service, Washington, DC, USA

“The man sure had a palate covered o’er / With brass or steel, that on the rocky shore / First broke the oozy oyster’s pearly coat / And risk’d the living morsel down his throat,” wrote John Gay (1685–1732), one of many poets since antiquity who became fascinated with the subject. More recently, Irish bard Seamus Heaney after enjoying fresh oysters with some friends, was so moved that he wrote a poem, “Laying down a perfect memory / In the cool of thatch and crockery,” and affirming the general idea that those of us who have never swallowed an oyster may not have lived life to the fullest. “Our shells clacked on the plates. / My tongue was a filling estuary, / My palate hung with starlight: / As I tasted the salty Pleiades / Orion dipped his foot into the water.”

As if the experience were not enough, Heaney offered a little history, “Over the Alps, packed deep in hay and snow,” he wrote, “the Romans hauled their oysters south to Rome.” The proper way of moving oysters from place to place has not changed much. Nor has the process of growing, harvesting, shucking, or eating oysters changed. “Alive and violated, / They lay on their bed of ice: / Bivalves: the split bulb / And philandering sigh of ocean / Millions of them ripped and shucked and scattered.”

Their silky texture and taste of the sea alone would have made oysters a popular food, not to mention their rich nutritional value and simple abundance. In the New World, Native Americans appreciated them, as did the invading Spaniards, even if only for the pearls. In the 1800s, consumption of the eastern oyster outpaced beef as a source of protein in some regions. In Louisiana, various ethnic groups settled in local parishes and contributed to the oyster industry. In the mid-1840s, fishermen started to gather seed oysters, plant them in favorable spots, and allow them to grow to market size in estuaries near the Mississippi River and in coastal areas farther west, creating one of the most successful oyster cultivation industries in the country. The modern harvesting processes came about in the early 1900s. As for readying oysters for market, despite attempts to mechanize the process, commercial oyster shucking remains the method of choice. Though experienced shuckers can glean large quantities of meat very quickly, efficiency comes at the expense of sound labor practices. Oyster shucking is marred, in the very least, by the monotony of processing and the cacophony of pounding blades.

This soul-testing occupation, labor-intensive and dangerous, usually in frigid environment and in the face of seemingly inexhaustible harvest, is what Catherine Howell captured in *Oyster Shuckers*, on this month’s cover. In this scene, painted in New Orleans (as inscribed on the upper left canvas), workers go at the task leaned over an overloaded bench. Their faces and clothes are sympathetically cast in broad impressionist strokes and lit from the window. Despite the need to handle each specimen separately and the pressure to deliver the oyster whole and the shell undamaged, this is an assembly line. Abject boredom marks the vacant faces. This is piece work―the more oysters shucked, the more money made.

Not much is known about Catherine Howell, other than she studied at the Art Students League of New York and the School of the Art Institute of Chicago. Nor is it known why she selected oyster shucking as the subject of this painting. But, a native of East Feliciana Parish, Louisiana, she was clearly aware of the oyster industry amidst the poverty of her times. She was also interested in local history, having coauthored in 1936 The Perfect Blend of the Old and the New: a Story of the Famous Vieux Carré [French Quarter] of New Orleans.

Along with many others, Howell took advantage of the Public Works of Art Project (December 1933 to June 1934), a program, part of the New Deal, set up to support artists during the Great Depression. First of its kind, this program affirmed the value of art as a legitimate occupation needed in reconstructing a society unhinged by economic catastrophe. “Work must be found for artists as well as for longshoremen.” Or as President Roosevelt’s relief administrator put it, “They’ve got to eat just like other people.” Applicants had to prove they were professional artists, and they had to pass a needs test. Most who took the job were young. After doing their work for the nation, they returned to local or regional occupations and remain mostly unknown today. The art they produced was for the most part conservative by modern standards, but at the time, “It was a revelation to many people in America that the country even had artists in it.”

The newly hired workers were encouraged to paint the contemporary American scene: the cities and countryside, harbors and sidewalks, factories and coal mines, farms and orchards, church halls and baseball fields of everyday people at work and play, the cotton pickers, the restaurant and mill workers. Along the same lines, Catherine Howell’s *Oyster Shuckers* was chosen by the Roosevelts to hang in the White House. In addition to supporting unemployed artists, the arts project aimed to improve the appearance of public buildings and embellish common areas, bringing to the local population pride in their surroundings. Among buildings that benefited in Howell’s area were 18 Louisiana Post Offices, which received murals. The program was not without its critics, who decried having taxpayer money used for decoration.

Government programs, whether for the advancement of art or the promotion of public health, are always under scrutiny, sometimes for their perceived frivolity but most often for their cost-effectiveness or economic fallout. In addition to immediate financial benefit and value as a morale booster, New Deal art has left behind a precious legacy, an artistic record of the times. “One hundred years from now,” President Roosevelt predicted, “my administration will be remembered for its art, not its relief.” The same philosophy could well apply to public health. U.S. Government-funded disease surveillance systems, which have grown swiftly in scope and sophistication, are providing data for immediate improvements in health. At the same time, by exploring the effects of disease, as well as virus evolution and structure, vaccination, and other disease prevention measures, they also increase understanding of problems that have puzzled us since the beginning of time.

Author and philosopher Pliny the Elder discussed Roman fondness for oysters at great length. The best, he maintained, were found at the mouths of rivers. “It is hardly possible to say enough about them, for they have held first rank as a table delicacy for a long time.” His compatriots generally ate oysters raw, sometimes served covered with snow, often in large quantities. Emperor Clodius Albinus, known for his gluttony, was said to consume 400 at one sitting. “Oysters must be permitted when wanted, but seldom, because they are cold and phlegmatic,” wrote Greek physician Anthimus in his cookbook On the Observance of Foods. “But if oysters smell, and anyone eat of them, he has need of no other poison.” Anthimus’ observation in the 6th century was remarkably astute, despite the generally unreliable association between spoilage and safety.

Consumption of raw seafood has a long and storied past, and so does gastrointestinal illness associated with some shellfish, especially raw or undercooked oysters. One reason is their filter-feeding nature, which allows them to passively concentrate bacteria and viruses; another is their minimal processing and cooking before consumption. Advances in laboratory techniques and epidemiologic methods have honed in on the specific causes of enteric diseases, long perceived to be primarily bacterial or unknown. Contaminated oysters are now frequently implicated in norovirus outbreaks across the globe.

Despite sewage control and improvements in hygiene, enteric diseases caused by contaminated food and water or spread from person to person remain far too common. In the United States, norovirus is the leading cause of gastroenteritis. Food and friendship aside, in the case of oysters, poetry must still reside in a balanced combination of pleasure and responsibility. And as during the Public Works of Art Project, a long-term solution may also lie in art, this time the art of isolating pathogenic agents and gathering surveillance data.
